# In situ evaluation of the bleaching efficacy of experimental hydrogen peroxide hydrogel with MnO‐doped Biosilicate activated by violet LED light

**DOI:** 10.1111/eos.70089

**Published:** 2026-03-31

**Authors:** Rafael Dascanio, Marina Trevelin Souza, Camila Siqueira Silva Coelho, Matheus Kury, Edgar Dutra Zanotto, Vanessa Cavalli

**Affiliations:** ^1^ Piracicaba Dental School Department of Restorative Dentistry University of Campinas (UNICAMP) Piracicaba São Paulo Brazil; ^2^ Vitreous Materials Laboratory (LaMaV) Department of Materials Engineering Federal University of São Carlos (UFSCar) São Carlos São Paulo Brazil; ^3^ Dental Research Division Paulista University (UNIP) São Paulo São Paulo Brazil

**Keywords:** Biosilicate, in situ bleaching, manganese oxide

## Abstract

Hydrogen peroxide (HP) at 35% is effective for dental bleaching but may reduce enamel mineral content and induce structural changes. This study developed a bleaching‐hydrogel containing 6%HP and manganese‐oxide–doped Biosilicate (MnO_BioS), activated by violet LED, under in situ conditions. Pigmented enamel blocks were attached to palatal devices (*n* = 12) and kept intraorally for 15 days, being removed only for bleaching sessions. Experimental groups were as follows: 35%HP (positive control) and 6%HP gel containing BioS or MnO_BioS (0/10 wt%), with or without violet LED irradiation. Bleaching consisted of three 30‐min sessions with 7‐day intervals. Color change (Δ*E*_00_), whiteness index (ΔWI_D_), color coordinates (ΔL*, Δa*, Δb*), surface hardness recovery (%SHR), and enamel inorganic content (CO_3_^2−^/PO_4_^3−^ ratio by Fourier‐transform infrared) were evaluated at baseline (*T*_0_) and after treatment (*T*_1_), with scanning electron microscopy analysis at *T*_1_. Data were analyzed using analysis of variance and Tukey's‐test (*α* = 0.05). 6%HP_MnO_BioS_LED showed the highest bleaching effectiveness (Δ*E*_00_ = 8.90 ± 1.18; ΔWI_D_ = 20.86 ± 3.26), surpassing 35%HP in ΔWI_D_ (15.53 ± 1.33) and showing comparable Δ*E*_00_ (6.48 ± 1.81). MnO_BioS groups increased enamel CO_3_^2−^/PO_4_^3−^ ratios after bleaching (0.475 ± 0.02; 0.466 ± 0.01) compared with baseline (0.431 ± 0.01; 0.429 ± 0.01; *p* < 0.05). Hardness recovery improved (%SHR = 4.14 ± 1.88; 5.45 ± 2.54), whereas it decreased in 35%HP (−1.32 ± 1.67) and 6%HP_LED (−2.60 ± 0.96). 6%HP hydrogel containing MnO_BioS activated by violet LED enhanced bleaching efficacy while preserving enamel mineral‐related parameters.

## INTRODUCTION

Hydrogen peroxide (HP) continues to be the preferred agent for dental bleaching procedures, despite its well‐documented adverse implications, such as the reduction in enamel mineral content and structural changes, including microcracks, porosity, and modifications in the crystallinity of the enamel surface [1, 2]. These changes compromise the enamel's mechanical properties, making it more vulnerable to wear and damage. In addition, bleaching with high concentrations of HP can result in cytotoxicity and increased dental sensitivity [3]. To minimize the risks associated with high HP concentrations, strategies such as the use of reduced concentrations [4], violet LED irradiation (405–410 nm) [5, 6], and pH control [1] have been adopted. However, even with these more controlled approaches, complications such as dental sensitivity, present in approximately 60% of patients, continue to occur, often associated with pulp damage [7], as well as imbalances in calcium/phosphate levels and demineralization, even under controlled pH conditions [1].

The need for multiple sessions with low concentrations of HP to achieve the desired results in dental bleaching has been a growing concern [8], especially when compared to faster procedures with higher concentrations of HP, which involve shorter application times [9]. As a strategy, the composition of bleaching gels has been modified to improve efficacy without increasing risks, such as incorporating additional oxidizing agents [10, 11]. In this context, the introduction of transition metals, such as manganese oxide (MnO), emerges as a promising strategy to accelerate the decomposition of HP, enhancing its bleaching action in a more efficient and controlled manner [12, 13]. Due to its catalytic activity, MnO promotes the decomposition of HP into highly reactive free radicals, such as the hydroxyl radical (•OH), which are essential for oxidizing dental stains and bleaching enamel [14]. Moreover, the presence of MnO in the bleaching agent contributes to reducing the required concentration of HP, allowing effective results with lower concentrations of HP.

Furthermore, the exploration of vitroceramics, such as Biosilicate, represents an innovative and promising approach for preserving the mineral content of dental enamel [15]. Initially introduced in dentistry for treating dental hypersensitivity [16], Biosilicate has proven effective in protecting enamel against damage caused by bleaching agents [17, 18]. When incorporated into the bleaching gel, Biosilicate acts as a mineralizing protector, forming complexes with enamel and helping to reduce structural damage that may be caused by HP during bleaching [19]. Additionally, the presence of Biosilicate in the bleaching gel may contribute to decreasing the diffusion of HP towards the pulp chamber, especially in teeth with early dental erosion lesions, where enamel integrity may be compromised [7].

Violet LED light can play a crucial role in dental bleaching by interacting effectively with the chromophores present in enamel and dentin [20]. It induces photochemical reactions that result in the excitation of dental extrinsic pigments, particularly in the conjugated carbon–carbon (C = C) bonds, which are responsible for light absorption in the yellowed and stained regions of the enamel [21]. Furthermore, violet LED light has the ability to interact with transition metals, enhancing the catalytic action of these compounds [22, 23]. The interaction between violet light and MnO can increase the catalytic potential of manganese oxide, accelerating the decomposition of HP.

A recent in vitro study synthesized Biosilicate doped with manganese oxide (MnO_BioS), and the results indicated that the gel containing a low concentration of HP associated with MnO_BioS and activated by violet LED light not only increased the gel's pH but also enhanced photocatalysis, HP decomposition, and bleaching efficacy and promoted greater surface hardness recovery (SHR), in addition to increasing the concentrations of CO_3_^2−^ and PO_4_^3−^ in the treated enamel [24]. Although these promising results were observed in vitro, it is essential to conduct further in situ investigations to assess the effects in a dynamic environment, considering factors such as salivary flow, oral microflora, brushing cycles, and interactions between whitening agents and dental structures.

This study proposes the analysis of a hydrogel containing 6%HP, incorporating BioS doped with MnO, activated by violet LED light (405–410 nm) in in situ conditions. In this context, the null hypotheses are as follows: (1) The addition of MnO_BioS in the hydrogel will not affect the bleaching potential of the 6%HP gel; (2) the addition of MnO_BioS in the hydrogel will not affect enamel hardness; (3) the addition of MnO_BioS in the hydrogel will not alter the concentration of calcium and phosphate in dental enamel after bleaching.

## MATERIAL AND METHODS

### Experimental design

This in situ study was conducted in accordance with the relevant institutional and national regulations, following the principles of the Declaration of Helsinki. The research protocol was reviewed and approved by the Research Ethics Committee of the Piracicaba Dental School, University of Campinas (FOP‐UNICAMP), under approval number CAAE 70649223.1.0000.5418, on April 10, 2024.

All participants were informed about the objectives and procedures of the study and signed a written informed consent form prior to enrollment. The privacy rights of human subjects were strictly observed throughout the research.

A double‐blind in situ study was conducted with experimental bleaching gels. Palatal devices containing sterilized enamel blocks (*n*  =  12/group) were used by 12 healthy volunteers [25] over a period of 15 days. The devices remained in the oral environment and were only removed for the professional bleaching treatment sessions. Enamel/dentin bovine discs were prepared and selected based on initial average color values (L* parameter) and microhardness (KHN). For the experimental treatments, the discs were stained with black tea and then randomly distributed among the groups (*n*  =  10/group), which were subjected to different treatments as follows:

- 35%HP: 35%HP gel (whiteness HP, FGM, Indústria e Comércio de Produtos Odontológicos, Joinville, PR, Brazil).
- 6%HP: Experimental 6%HP gel.
- 6%HP_MnO_BioS: Experimental 6%HP gel + MnO‐doped Biosilicate (BioS).
- 6%HP_LED: Experimental 6%HP gel irradiated with violet LED.
- 6%HP_MnO_BioS_LED: Experimental 6%HP gel + MnO‐doped BioS irradiated with violet LED.

**FIGURE 1:**
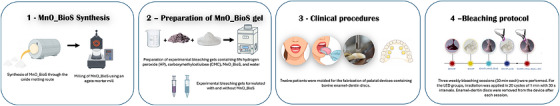
Schematic representation of the experimental design.

Figure 1 presents a schematic representation of the experimental design. The bleaching treatments were carried out in three sessions of 30 min each, on the 1st, 7th, and 14th days of treatment. The blocks were evaluated for color change (Δ*E*_00_), bleaching index (ΔWI_D_), and color parameters (L*, a*, b*) using a digital spectrophotometer, and mineral content (surface microhardness [KHN] and evaluation of carbonate [CO_3_^2−^] and phosphate [PO_4_^2−^] bands using Fourier‐transform infrared [FTIR] spectroscopy). The color, microhardness, and mineral content (FTIR) analyses were performed after pigmentation (baseline, *T*_0_) and 24 h after the last bleaching session (*T*_1_). Enamel morphology was evaluated by scanning electron microscopy (SEM) at the end of the treatment (*T*_1_).

### Biosilicate synthesis

The MnO_BioS was synthesized using the oxide fusion route [15], with the addition of 2.5 mg/mL of manganese oxide (MnO). The synthesis resulted in the composition 24.3Na_2_O–26.9(*x*CaO‐(1 − *x*)MnO)–46.3SiO_2_–2.5P_2_O_5_ (*x*  =  1 and 0.9, in weight %). First, the raw materials (CaCO_3_, Na_2_CO_3_, SiO_2_, NaH_2_PO_4_, and MnO) were mixed and homogenized in a jar mill for 24 h. Then, the glass fusion process was carried out in a platinum crucible in a bottom‐load furnace (Nabertherm, DE) at 1450°C until complete fusion and the formation of a homogeneous glass. After fusion, the molten material was rapidly cooled, resulting in the formation of a glassy phase. Subsequently, the glass cylinders were ground in a planetary mill (Fritsch, DE) to achieve an average particle size of 5 µm (d50). Cylinders of MnO_BioS with a diameter of 12 mm and a height of 50 mm were obtained, and these were ground in a planetary mill using an agate jar and grinding media [24].

### Gel preparation

The bleaching gel used as a control was 35% HP (Whiteness HP, FGM, Indústria e Comércio de Produtos Odontológicos, Joinville, PR, Brazil), prepared according to the manufacturer's instructions. To formulate the 6%HP gel, a dilution of 35%HP was made and then combined with a thickening gel based on carboxymethylcellulose (CMC), using a proportion of 0.4 g of thickener for 0.54 mL of the 6%HP solution. The mixture was homogenized in a Speed Mixer (DAC Iso 1. FVZ, FlackTek) at 2000 rpm for 2 min. For the experimental gels containing MnO_BioS, the thickener CMC and distilled water (H_2_Od) were used [7]. The MnO_BioS particles were prepared by the conventional fusion method and incorporated into the CMC gel and homogenized in the Speed Mixer (DAC Iso 1. FVZ, FlackTek) for 2 min at 2000 rpm. Subsequently, 0.4 g of this gel was mixed with 0.54 mL of 6%HP solution [24].

### Preparation of specimens

Bovine teeth with intact enamel, free of fractures or cracks, were carefully cleaned and disinfected with a 0.5% thymol solution (Labsynth). Discs of enamel and dentin, with a diameter of 5 mm and thickness of 3 mm, were prepared using a bench drill (FSB 16, Pratika, Shulz) and core drills. The dentin surface was initially leveled in a polishing machine (AROTEC) with a #600 sandpaper to ensure parallelism of the samples. The enamel was then abraded with silicon carbide sandpapers (#600, 800, 1200, 3 M ESPE 411Q) and polished with a felt disk and diamond suspension (abrasive particles of 1 and ½ µm) for 2 min, standardizing the final thickness of the samples at 3 mm, as described in previous studies [7].

### Staining with black tea

The bovine tooth specimens were immersed in a black tea solution following ‐a protocol previously described [24, 25]. To prepare the solution, 2 g of black tea was dissolved in 100 mL of distilled water for 5 min. After filtering and buffering the solution, the specimens were kept in it for 48 h in a 37°C incubator. The specimens were then transferred to artificial saliva, composed of 1.5 mM Ca^2+^, 0.9 mM PO_4_^3−^, 150 mM KCl, and 20 mM tris buffer at pH 7.0, and remained for 7 days to stabilize the color before the treatments. The artificial saliva was renewed every 3 days [24]. After pigmentation, enamel blocks that exhibited L* (luminosity) and KHN (microhardness) values with variations of up to 10% from the overall average for all specimens were selected [5].

### Volunteers’ selection

Δ*E*_00_ was considered the primary outcome and an 80% power (*β*) and a 5% level of significance (*α*) were adopted. Using g*power software, a minimal detectable difference of 0.8 Δ*E*_00_ units, and a standard deviation of 0.56 derived from a previous in situ study [26] were set. Under these assumptions, approximately 10 participants were required. To account for potential dropouts and to ensure adequate power for comparisons among the experimental groups, 12 participants were included in the present study. The participants were undergraduate and graduate students from the dentistry course at FOP/UNICAMP. After the initial screening, the following inclusion criteria were applied: good general and oral health, no antibiotic treatment in the last 30 days, non‐smokers, no use of medications affecting salivary flow, and no history of previous whitening treatments, either at home, in the office, or with over‐the‐counter whitening products such as toothpaste, mouthwashes, whitening strips, prefabricated trays, or blue or violet light devices. Volunteers who did not meet these requirements were excluded from the study. Before starting the study, participants were given the informed consent form (ICF) and an initial medical history questionnaire. This study was approved by the Ethics Committee (CAAE: 70649223.1.0000.5418) at the Universidade Estadual de Campinas—UNICAMP.

### In situ experimental phase

Palatal devices were manufactured from alginate impressions of the patient's dental arches using transparent acrylic resin. The specimens were attached to the palatal device using sticky wax and removed for the stablished treatments and analyses [26]. Each device contained six cylindrical dental blocks, which were subjected to the bleaching treatments as per the established groups.

Randomization was performed at the disc level prior to insertion into the palatal appliances using a computer‐generated random sequence. For each volunteer, enamel discs representing all experimental groups were randomly allocated to predefined positions within the same appliance, ensuring that each subject served as their own control. The spatial distribution of the blocks was balanced to avoid systematic positional bias within the appliance. To minimize potential intra‐volunteer clustering effects, the experimental design intentionally exposed all treatment groups to the same oral environment (saliva composition, flow rate, temperature, and oral hygiene habits) within each volunteer. As a result, comparisons were primarily performed between treatment conditions under identical intraoral conditions rather than between volunteers. This split‐mouth–like in situ design is widely adopted in palatal appliance studies and is considered an effective strategy to control inter‐individual variability.

The 12 volunteers used the devices, which were removed only during meals on the scheduled days for the whitening treatment (1st, 7th, and 15th days) and for brushing. To ensure standardized brushing, the participants were given fluoride toothpaste (1450 ppmF, Colgate Total 12, Colgate‐Palmolive) and a soft‐bristled toothbrush. They were instructed to brush the dental blocks twice a day, for 1 min each time, using circular movements (in the morning and at night), in addition to regular brushing three times a day with the same toothpaste. On the days designated for the whitening treatments, the volunteers attended the laboratory and handed over the devices to the researcher, who then began the whitening treatment according to the protocols described below. After each bleaching session, the devices were returned to the volunteers. On the 15th day, the devices were collected for the evaluation of color change (Δ*E*_00_), whiteness index (ΔWI_D_), color parameters (L*, a*, b*). Besides, the %SHR, the concentration of carbonate (CO_3_^2−^) and phosphate (PO_4_^3−^), and enamel surface morphology were determined by microhardness test, FTIR, and SEM, respectively. Double blinding was ensured by separating the bleaching procedures from both the participants and the outcome assessor. Volunteers did not accompany the bleaching procedures or any laboratory tests, as all enamel blocks were removed from the palatal appliances and treated outside the oral environment. Therefore, participants had no visual or procedural contact with the bleaching protocols, gel characteristics, or LED activation. All outcome assessments were performed by a trained evaluator who did not participate in the bleaching procedures and had no contact with the volunteers. Enamel specimens were coded by an independent researcher before the analysis, and group allocation was disclosed only after completion of all measurements and statistical analyses.

### Bleaching protocol

The bleaching protocol was conducted as described by Kury *et al*. [27]. The bleaching gels were applied to the entire enamel surface in a layer approximately 1 mm thick, followed by rinsing with purified water. This procedure was performed over three sessions, with intervals of 7 days between each session. During each session, only one application of the experimental bleaching gel (with or without the combination with bioactive glasses) was carried out, with or without the irradiation of violet LED light, according to the experimental groups. The LED light was applied in 20 cycles of 1 min each, with 30‐s intervals between cycles (405 ± 10 nm, 1.2 W/cm^2^, emission area  =  10.7 cm^2^, Bright Max Whitening, MMO, São Carlos, SP, Brazil). For the bleaching protocol, the specimens were removed from the intraoral device. For the LED groups, the specimens were positioned equidistantly from the light source to ensure standardized irradiance across all samples.

### Color analysis

The color parameters L* (black–white axis), a* (red–green axis), and b* (yellow–blue axis) were assessed using a digital spectrophotometer (EasyShade, Vita Zahnfabrik). The specimens were positioned on an opaque white ceramic tile to standardize the background and minimize light interference. The Easyshade spectrophotometer was secured in a fixed holding device to ensure consistent probe positioning and angulation throughout all measurements. Color assessments were performed inside an artificial light cabin with standardized illumination conditions to eliminate ambient light variability. The Δ*E*_00_ and ΔWI_D_ values were measured after staining with black tea (*T*_0_) and 24 h after the last bleaching session (*T*_2_). The Δ*E*_00_ values were evaluated based on the perception threshold (PT) and acceptance threshold (AT), set at 0.81 and 1.8 U, respectively. Similarly, the variation in the whiteness index (ΔWI_D_) was compared to the PT and AT thresholds, which were defined as 0.7 and 2.6, respectively. The color variation was calculated using the CIEDE2000 formula (Δ*E*_00_) [28]:

$$\Delta E_{00}=\sqrt{\begin{array}{ccc} {\left(\frac{\Delta L^{\prime }}{k_{L}S_{L}}\right)}^{2}+ & {\left(\frac{\Delta C^{\prime }}{k_{C}S_{C}}\right)}^{2}+ & \begin{array}{ccc} {\left(\frac{\Delta H^{\prime }}{k_{H}S_{H}}\right)}^{2}+\mathrm{RT} & \left(\frac{\Delta C^{\prime }}{k_{C}S_{C}}\right) & \left(\frac{\Delta H^{\prime }}{k_{H}S_{H}}\right) \end{array} \end{array}}$$

The variation in the whiteness index was calculated using the following formula:

$${\mathrm{WI}}_{D}=\left[\left(0.511\times L*\right)-\left(2.324\times a*\right)-\left(1.100\times b*\right)\right]$$

### Carbonate (CO_3_^2−^) and phosphate (PO_4_^3−^) concentration in enamel

Infrared spectra of bovine enamel were collected at three separate points on each sample, both prior to (*T*_0_) and following the treatments (*T*_1_), using a FTIR spectrometer (Nicolet IS50, Thermo Fisher). The instrument was set to scan within the range of 500–4500 cm^−1^, with a resolution of 4 cm^−1^, and each spectrum/location was averaged over 10 scans. An attenuated total reflectance (ATR) accessory with a monolithic diamond crystal (Golden Gate, Specac) was attached to the spectrometer. The specimens were positioned on the ATR crystal at the same predefined three locations at both *T*_0_ and *T*_1_. A reference notch was created at the analysis’ spots of the specimen on the side opposite to the enamel surface to ensure consistent positioning of the pressure knob during all measurements. Any surface changes detected in the enamel were inherent to the treatment applied and represented the intended outcome of the analysis [24].

The spectral data were processed by correcting for water content, applying a baseline adjustment, and normalizing using OMNIC software (v7.0). After treatment (*T*_1_), the areas under the peaks associated with CO_3_^2−^ *v*_2_ (886 cm^−1^), PO_4_^3−^ *v*_1_ (996 cm^−1^), and PO_4_^3−^ *v*_2_ (1410–1460 cm^−1^) were calculated. The enamel's mineral composition, defined as the carbonate‐to‐phosphate ratio, was determined by integrating the areas under the CO_3_^2−^ *v*_2_ and PO_4_^3−^ (*v*_1_ and *v*_2_) peaks [24].

### Surface hardness recovery percentage (%SHR)

The enamel blocks’ surface microhardness was determined by making three indentations in the central region of each block, using a Knoop indenter (Future Tech‐FM‐1e). A static load of 25 g was applied for 5 s, with a spacing of 100 µm between each indentation. Microhardness evaluations were conducted after staining (*T*_0_) and 24 h following the final bleaching session (*T*_1_). To evaluate the immediate remineralizing effect of the experimental agents, the percentage of SHR was calculated using the following equation:

$$\begin{array}{ccc} & & \%\mathrm{SHR}=\\ & & \left(\frac{\mathrm{Microhardness}\;\mathrm{after}\;\mathrm{treatment}\;-\;\mathrm{Initial}\;\mathrm{microhardness}}{\mathrm{Initial}\;\mathrm{microhardness}}\right)\\ & & \times 100 \end{array}$$

### Scanning electron microscopy (SEM)

Morphological analysis was performed using SEM on three representative samples from each group after the third bleaching session (*T*_1_). The samples were cleaned in an ultrasonic bath (Ultra Cleaner, Unique) for 10 min, followed by drying in an oven for 24 h. Once dried, the samples were coated with carbon and analyzed using a SEM (JEOL‐JSM, 6460LV), equipped with an automated image acquisition system. The microscope operated at 15 kV under vacuum mode, and representative images were captured at a magnification of 1500× [29].

### Statistical analysis

The data were subjected to statistical analysis using SPSS Statistics Version 23 (IBM Corp). Normal distribution and homoscedasticity were verified, and then the data were analyzed using one‐way analysis of variance (anova) followed by Tukey's post hoc test. For calcium and phosphate concentrations, the data were analyzed using repeated measures anova followed by Tukey's post hoc test.

## RESULTS

### Color change analysis

**FIGURE 2:**
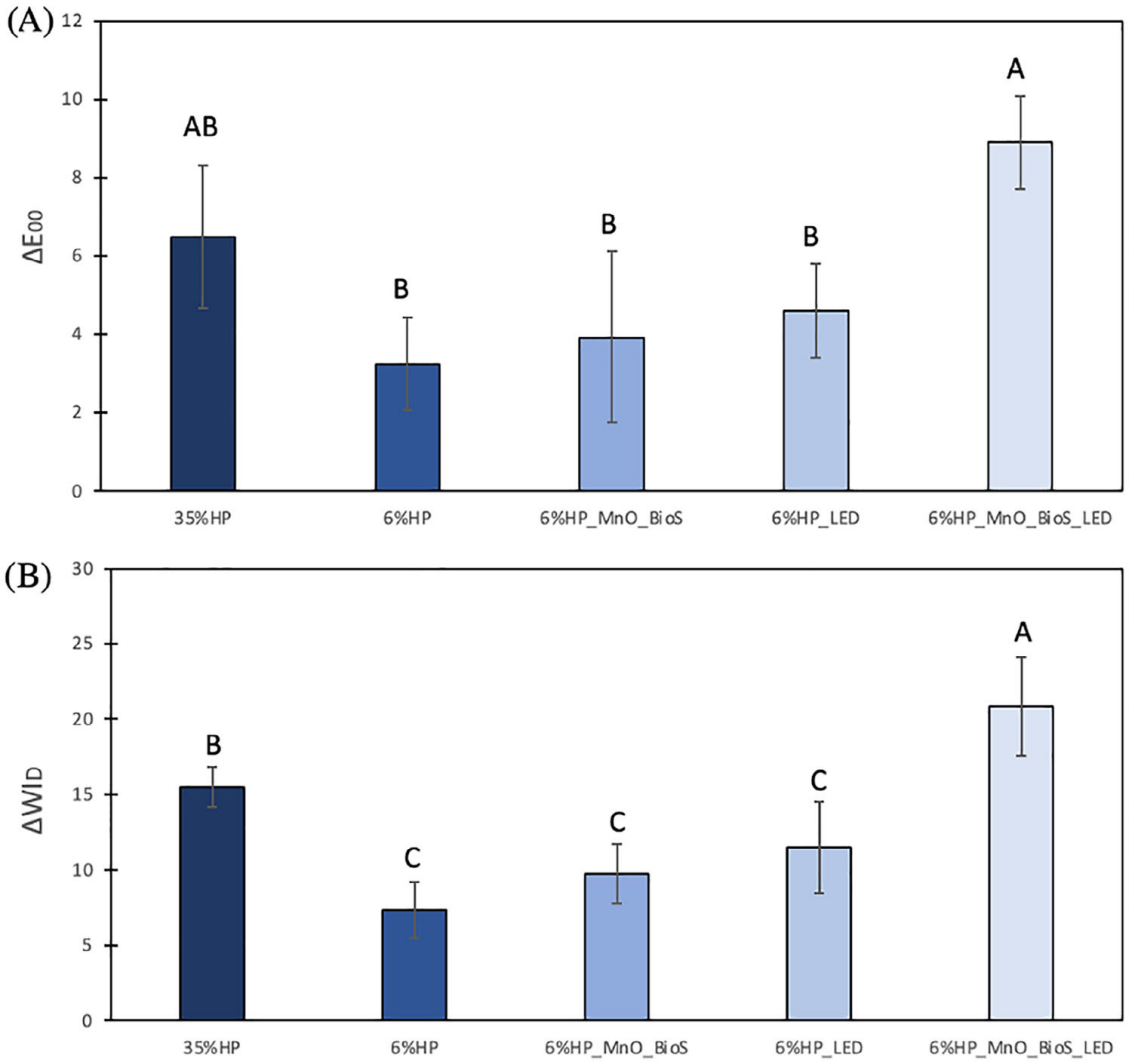
Mean and standard deviation of Δ*E*_00_ (A) and ΔWI_D_ (B) considering the values collected at baseline (*T*_0_) and 24 h after the last bleaching session (*T*_1_). Means followed by different letters indicate statistical differences according to one‐way anova and Tukey's test (*p* < 0.05).

Figure 2A and Table 1 display the graph of color variation (Δ*E*_00_). 6%HP_MnO_BioS_LED group demonstrated the greatest color change, with significant differences when compared to the other groups (*p* < 0.05). The 35%HP group showed intermediate values (*p* < 0.05) but did not differ from the 6%HP, 6%HP_MnO_BioS, and 6%HP_LED groups, which exhibited the lowest color change indices, with no significant differences among them (*p* > 0.05). Figure 2B and Table 1 show the graph of the variation in the whiteness index (ΔWI_D_). The 6%HP_MnO_BioS_LED group achieved the highest bleaching values, with a statistically significant difference compared to the other groups (*p* < 0.05). The 35%HP group showed intermediate values, also with a significant difference when compared to the 6%HP, 6%HP_MnO_BioS, and 6%HP_LED groups, which presented the lowest bleaching indices (*p* < 0.05).

**TABLE 1:** Color difference (Δ*E*_00_) and whiteness index (ΔWI_D_) after bleaching (*T*_1_–*T*_0_).

Group	Δ*E*_00_	ΔWI_D_
35%HP	6.48 ± 1.81AB	15.53 ± 1.33B
6%HP	3.24 ± 1.19B	7.36 ± 1.83C
6%HP_MnO_BioS	3.92 ± 2.18B	9.75 ± 1.96C
6%HP_LED	4.59 ± 1.20B	11.53 ± 3.03C
6%HP_MnO_BioS_LED	8.90 ± 1.18A	20.86 ± 3.26A

*Note*: Values are expressed as mean ± standard deviation. Different uppercase letters indicate statistically significant differences among groups (anova /Tukey, *p* < 0.05). Δ*E*_00_  =  CIEDE2000 color difference; ΔWI_D_  =  whiteness index for dentistry.

**FIGURE 3:**
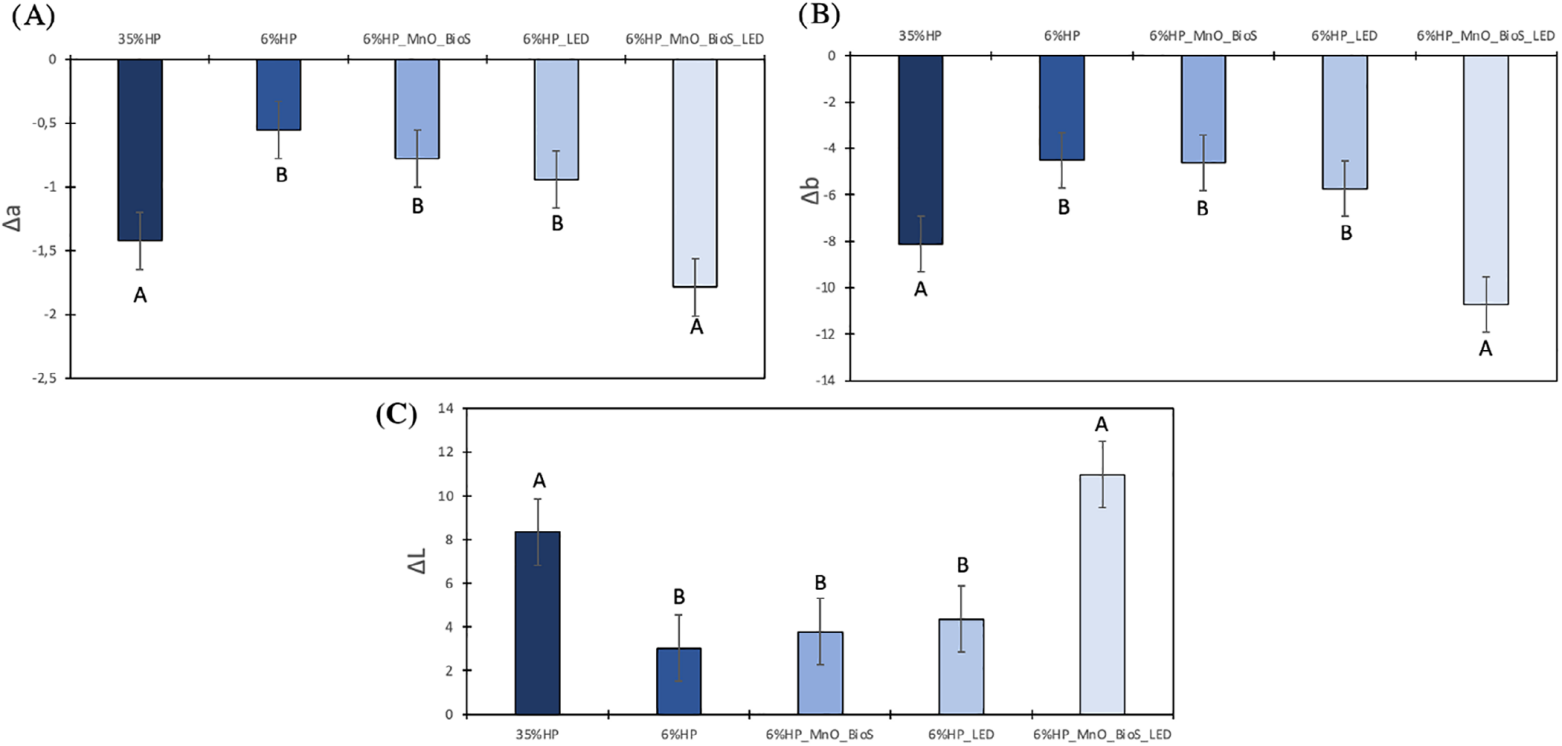
Mean and standard deviation of Δ*a* (A), Δ*b* (B), and Δ*L* (C) considering the values collected (*T*_0_) and 24 h after the last bleaching session (*T*_1_). Means followed by distinct letters indicate statistical differences according to one‐way anova and Tukey's test (*p* < 0.05).

The variation of the a* axis (Δ*a*) is presented in Figure 3A and Table 2. No significant differences were observed between the 35%HP and 6%HP_MnO_BioS_LED groups (*p* > 0.05), with both showing Δ*a* values higher than those of the 6%HP, 6%HP_MnO_BioS, and 6%HP_LED groups, which exhibited significantly lower Δ*a* values (*p* < 0.05). In Figure 3B and Table 2, regarding the variation of the b* axis (Δ*b*), the results were similar: the 35%HP and 6%HP_MnO_BioS_LED groups did not differ statistically from each other (*p* > 0.05), but both showed higher Δ*b* values compared to the 6%HP, 6%HP_MnO_BioS, and 6%HP_LED groups, whose Δ*b* values were significantly lower (*p* < 0.05). The variation of the L* axis (Δ*L*), shown in Figure 3C and Table 2, followed the same trend. No significant difference was found between the 35%HP and 6%HP_MnO_BioS_LED groups (*p* > 0.05), with both showing higher Δ*L* values than the 6%HP, 6%HP_MnO_BioS, and 6%HP_LED groups (*p* < 0.05).

**TABLE 2:** Color coordinates variation (Δ*L*, Δ*a*, Δ*b*) after bleaching (*T*_1_–*T*_0_).

Group	ΔL*	Δa*	Δb*
35%HP	8.33 ± 2.83A	−1.43 ± 0.59A	−8.12 ± 1.65A
6%HP	3.03 ± 2.20B	−0.56 ± 0.48B	−4.50 ± 0.86B
6%HP_MnO_BioS	3.80 ± 2.00B	−0.78 ± 0.62B	−4.62 ± 2.56B
6%HP_LED	4.37 ± 2.06B	−0.94 ± 0.59B	−5.75 ± 2.86B
6%HP_MnO_BioS_LED	10.97 ± 3.50A	−1.79 ± 0.68A	−10.73 ± 4.02A

*Note*: Values are expressed as mean ± standard deviation. Different uppercase letters indicate statistically significant differences among groups (anova /Tukey, *p* < 0.05).

### Concentration of carbonate (CO_3_^2−^) and phosphate (PO_4_^2−^) in enamel

**FIGURE 4:**
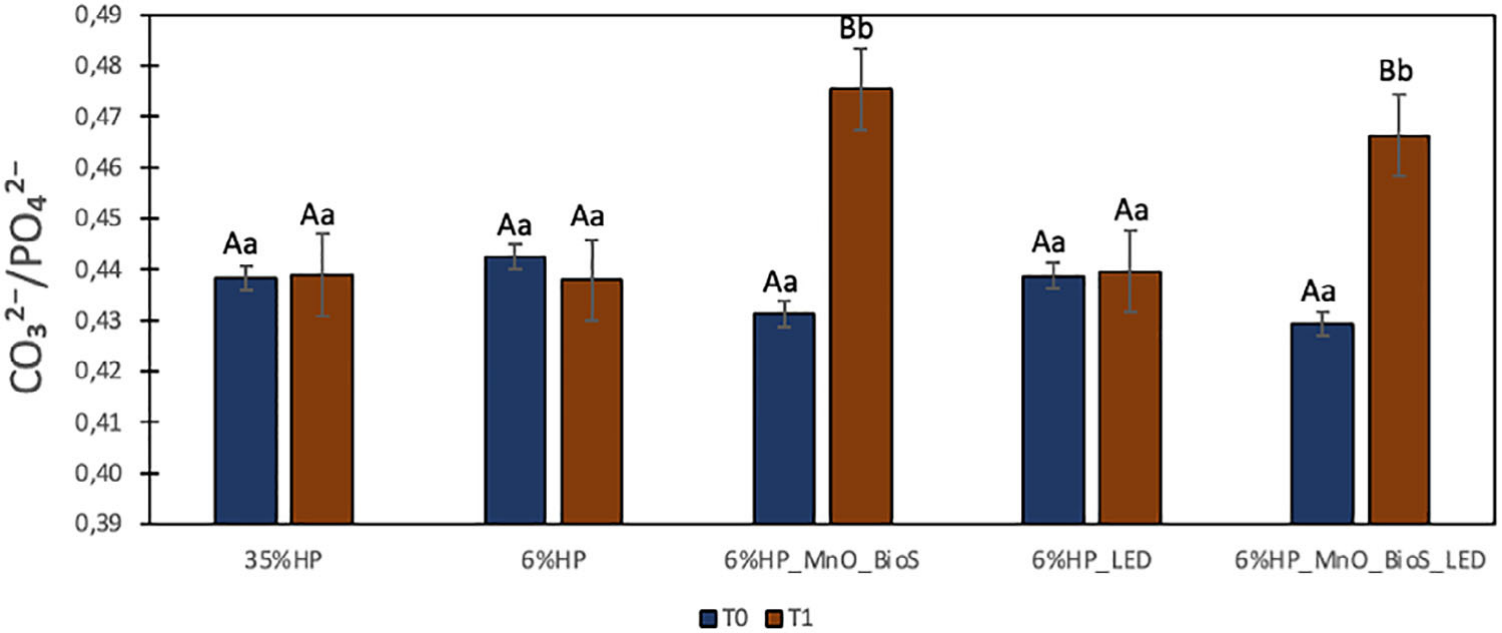
Values and standard deviations of the carbonate and phosphate (CO_3_^2−^/PO_4_^2−^) bands observed by Fourier transform infrared spectroscopy (FTIR) at times *T*_0_ (blue) and *T*_1_ (orange). Different uppercase letters indicate statistical differences within the same time point. Different lowercase letters indicate statistical differences between different time points, as determined by two‐way ANOVA and Tukey's test.

Figure 4 and Table 3 display the concentration of carbonate (CO_3_^2−^) and phosphate (PO_4_^2−^) in enamel. The groups treated with gels containing MnO_BioS showed a significant increase in the concentration of carbonate (CO_3_^2−^) and phosphate (PO_4_^2−^) after the third bleaching session (*p* < 0.05), regardless of the presence of LED light (*p* < 0.05). On the other hand, the groups without MnO_BioS maintained their carbonate (CO_3_^2−^) and phosphate (PO_4_^2−^) concentrations after bleaching (*p* > 0.05).

**TABLE 3:** Surface hardness recovery (%SHR) after bleaching (*T*_1_–*T*_0_).

Group	%SHR
**35%HP**	−1.32 ± 1.67B
**6%HP**	−0.72 ± 1.00B
**6%HP_MnO_BioS**	4.14 ± 1.88A
**6%HP_LED**	−2.60 ± 0.96B
**6%HP_MnO_BioS_LED**	5.45 ± 2.54A

*Note*: Values are expressed as mean ± standard deviation. Different uppercase letters indicate statistically significant differences among groups (one‐way anova /Tukey, *p* < 0.05).

### Surface hardness recovery (%)

**FIGURE 5:**
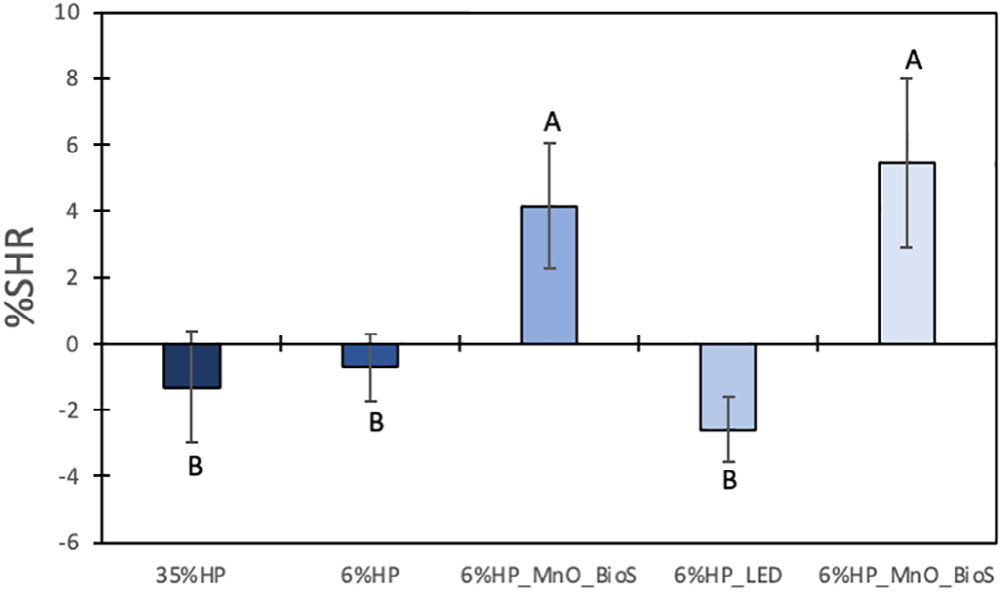
Percentage of surface hardness recovery (%SHR) values and standard deviation obtained (*T*_1_–*T*_0_). Different letters indicate statistical differences, according to one‐way anova and Tukey's test.

The surface hardness data from the groups subjected to bleaching were used to calculate the %SHR (Figure 5 and Table 4). The incorporation of MnO_BioS into the bleaching gel resulted in a 5% increase in %SHR, significantly differing from the groups containing only 35%HP or 6%HP, with or without LED irradiation, which showed a decrease in %SHR. The lowest %SHR values among the groups were observed in 35%HP (−1.32%), 6%HP (−0.72%), and 6%HP_LED (−2.59%), whereas 6%HP_MnO_BioS (4.14%) and 6%HP_MnO_BioS_LED (5.45%) demonstrated a greater recovery of enamel hardness (SH), regardless of LED irradiation (*p* > 0.05).

**TABLE 4:** Fourier‐transform infrared (FTIR) carbonate/phosphate ratio (CO_3_^2−^/PO_4_^3−^) before and after bleaching (*T*_0_ and *T*_1_).

	FTIR	
Groups	Before	After
**35%HP**	0.438 ± 0.01Aa	0.438 ± 0.01Aa
**6%HP**	0.442 ± 0.01Aa	0.437 ± 0.01Aa
**6%HP_MnO_BioS**	0.431 ± 0.01Aa	0.475 ± 0.02Bb
**6%HP_LED**	0.438 ± 0.01Aa	0.439 ± 0.01Aa
**6%HP_MnO_BioS_LED**	0.429 ± 0.01Aa	0.466 ± 0.01Bb

*Note*: Values and standard deviations of the carbonate and phosphate (CO_3_^2−^/PO_4_^2−^) bands observed by Fourier transform infrared spectroscopy (FTIR) at times *T*_0_ and *T*_1_. Different uppercase letters indicate statistical differences within the same time point. Different lowercase letters indicate statistical differences between different time points, as determined by two‐way anova and Tukey's test.

### Scanning electron microscopy (SEM)

**FIGURE 6:**
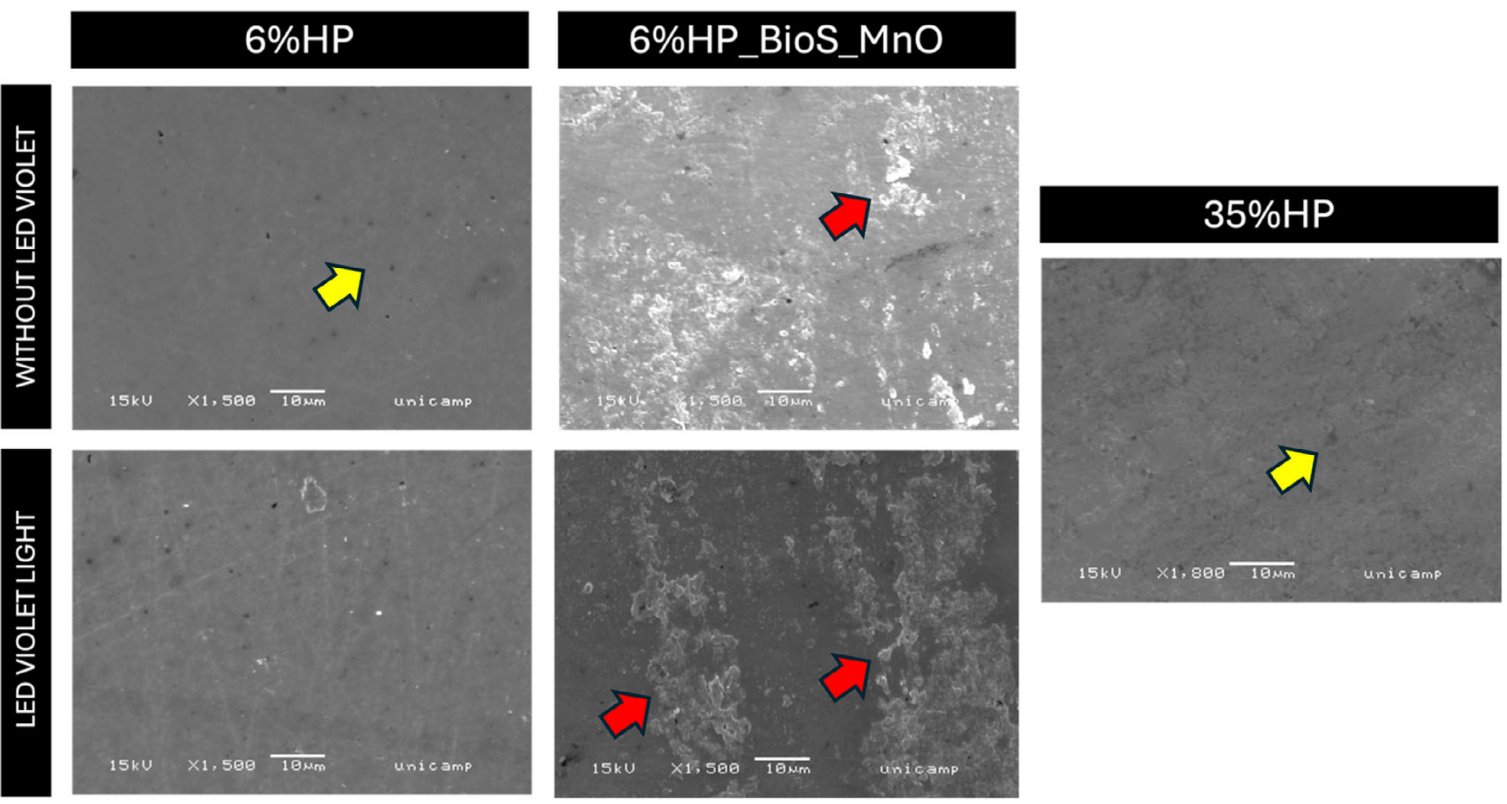
Scanning electron microscopy (SEM) of the treated groups. The yellow arrows indicate areas with surface roughness, suggesting morphological changes in the enamel. The red arrows highlight areas indicative of particulate deposition of BioS or MnO_BioS, demonstrating the formation of a protective layer.

The SEM images (Figure 6) revealed that the groups treated with MnO_BioS, with or without violet LED irradiation, exhibited enamel surfaces with characteristics suggesting the formation of a protective layer, possibly resulting from the particulate deposition of BioS, as indicated by the red arrows. In contrast, the groups without bioactive compounds (35%HP and 6%HP) displayed extensive areas of roughness, indicated by the yellow arrows.

## DISCUSSION

The Δ*E*_00_ results indicated that the addition of MnO_BioS to the 6%HP gel increased the bleaching effectiveness, especially when the gel was irradiated with violet LED light. The Δ*E*_00_ values observed for 6%HP_MnO_BioS_LED were similar to those obtained for the 35%HP gel, confirming that the addition of MnO_BioS enhanced the bleaching effectiveness of the low‐concentration gel. Additionally, the groups containing 6%HP (Δ*E*_00_  =  3.23) and 6%HP_MnO_BioS (Δ*E*_00_  =  3.92) exceeded the perceptibility threshold (PT > 0.8) and acceptability threshold (AT > 1.8) [28]. Furthermore, the positive ΔWI_D_ values reinforce the bleaching potential of 6%HP_MnO_BioS_LED, which showed a statistically significant difference compared to the other groups. These results indicate that the presence of MnO_BioS in the gel significantly contributed to the improvement of bleaching, rejecting the first null hypothesis, which suggested that the addition of MnO would have no effect on bleaching effectiveness. In vitro studies incorporating MnO_BioS into the low‐concentration gel also found no significant difference compared to 35%HP [24], corroborating the findings of our study, which suggest that even in an in situ situation, with the challenges of brushing and microbiota, bleaching with MnO_BioS proved effective. This result is further supported by the evaluation of the L* (lightness), a* (green–red axis), and b* (yellow–blue axis) parameters, where no significant differences were observed between 35%HP and 6%HP_MnO_BioS_LED.

The chromophores present in enamel and dentin are mainly organic compounds that absorb light in specific bands of the visible spectrum. The pigments responsible for tooth discoloration can be classified as extrinsic or intrinsic. Extrinsic stains originate from dietary sources, smoking, and environmental factors, leading to the accumulation of organic pigments on the enamel surface. In contrast, intrinsic discoloration results from aging and metabolic changes that affect the dentin structure over time [20]. They have absorptive characteristics, especially in the wavelength ranges between 380 and 420 nm. The violet LED light operates in the visible spectrum between 405 and 410 nm in two main ways: photochemical and photophysical. Photochemical activity involves the promotion of direct chemical reactions in association with the bleaching gel, whereas photophysical activity refers to the interaction of light with particles and molecules in the system, without generating immediate chemical reactions [21]. Photophysical activity can alter the energy of electrons and the structure of chromophore molecules in a non‐permanent way, which also contributes to bleaching [20]. When absorbed by chromophore molecules in dental enamel, violet light excites the electrons of these molecules to higher energy states, a process known as electronic excitation. After this excitation, the molecules return to their lower energy state (ground state), releasing the excess energy in the form of heat or fluorescence [21]. This process can also alter the molecular structure, facilitating the breakdown of chromophores. The violet LED light has been associated with the breaking of chemical bonds in extrinsic pigments, particularly the double bonds (C = C), which are susceptible to photolysis. This mechanism contributes to the discoloration of stains and the aesthetic improvement of dental enamel. However, this explanation mainly applies to studies that evaluate the effect of light in isolation. Clinically, LED light alone does not produce a significant whitening effect that would justify the increased efficacy observed in HP + LED groups solely through photophysical and photochemical processes [20, 21]. When applied, the violet light intensifies the photochemical activity of HP and other bleaching agents, promoting the release of molecular oxygen (O_2_) and free radicals [30]. These radicals, such as the hydroxyl radical (•OH) and the superoxide radical (O_2_•^−^), are highly reactive and can break the double bonds present in chromophore molecules, resulting in the discoloration of the pigments responsible for dental stains. The breaking of double bonds decreases light absorption in the yellowed areas, generating the visible bleaching effect [31].

However, violet LED light irradiation alone was not able to increase the bleaching effectiveness of the low‐concentration gel (6%HP). In contrast, the combination of MnO_BioS and violet LED light did enhance the bleaching effectiveness, as corroborated by in vitro studies [24]. The addition of MnO_BioS acts as a catalyst, primarily due to the redox capacity of MnO, promoting reactions similar to Fenton reactions with HP [29]. These reactions generate highly reactive radicals, such as the hydroxyl radical (•OH), which play an essential role in the decomposition of HP and dental bleaching. When MnO is oxidized from Mn^2+^ to Mn^3+^, it catalyzes the breakdown of HP into water (H_2_O) and oxygen (O_2_) [32]. This process accelerates the decomposition of peroxide, preventing it from remaining on the dental surface for an extended period, where it could generate reactive oxygen species harmful to pulp cells. In vitro studies using MnO_BioS with violet LED light showed greater efficacy than 35%HP in decomposing solutions containing methylene blue, an indicator of the bleaching ability of the solution to degrade pigments [24], which justifies the observed increase in bleaching effectiveness and corroborates the results obtained. Additionally, the acceleration of HP decomposition by MnO's action increases the release of molecular oxygen (O_2_), which, although non‐toxic, contributes to the oxidation of organic stains present in dental enamel [32]. Violet light plays a crucial role in this process, activating Mn^2+^/Mn^3+^ compounds of MnO and promoting greater efficiency in Fenton reactions [22, 23]. With this activation, a more intense production of free radicals occurs, such as hydroxyl radicals (•OH), which are particularly effective in removing organic stains and decomposing HP molecules, resulting in more efficient bleaching.

The %SHR results and the concentrations of carbonate and phosphate indicate that the addition of MnO_BioS to the bleaching gel increased the %SHR, with a statistically significant difference compared to 35% (HP35) or 6% (HP6) HP, both irradiated and non‐irradiated by LED, which showed a decrease in %SHR. After the third bleaching session, the groups treated with gels containing BioS or MnO_BioS showed a significant increase in carbonate and phosphate (CO_3_^2−^/PO_4_^3−^) concentrations, regardless of the LED light irradiation. The significant increase in %SHR and CO_3_^2−^/PO_4_^3−^ in the groups treated with MnO_BioS is consistent with in vitro study in the literature, which observed an increase in %SHR and carbonate concentrations with the use of BioS‐containing hydrogel, with or without the addition of MnO [24]. These findings can be explained based on the interaction steps of BioS with the aqueous medium and the release of calcium and amorphous phosphate.

As this was an in situ study, it was possible to directly evaluate the interaction of MnO_BioS with the oral cavity and with human saliva, providing a more realistic view of how these compounds act in a physiological environment. MnO_BioS is composed of a bioactive matrix that releases ions such as calcium (Ca^2+^), sodium (Na^+^), and phosphate (PO_4_^3−^) when in contact with saliva or other aqueous fluids [33]. When the gel containing MnO_BioS is applied to dental enamel, the released ions initiate an ion exchange with the hydrogen ions (H^+^) present in the HP‐containing gel, modifying the composition of the solution and creating a supersaturated environment with mineral ions [34]. This ionic release process favors the formation of a protective calcium phosphate (Ca_3_(PO_4_)_2_) rich layer on the enamel surface, which may help restore the mineralization of the dental structure. The exchange of sodium ions (Na^+^) for hydrogen (H^+^) or hydronium (H_3_O^+^) ions creates a supersaturated ionic reservoir, highly saturated with calcium and phosphate, favoring the nucleation of minerals, such as amorphous calcium phosphate (ACP), a precursor to the formation of apatite [32]. After the partial dissolution of MnO_BioS, the silanol groups (Si–OH) present in the bioactive matrix reorganize and act as nucleation centers for the ACP phases [15]. The undissolved MnO_BioS particles, along with the calcium and phosphate ions present in the solution, facilitate the formation of this protective layer on dental enamel. This mineralized layer acts as a barrier against further acid attacks, preserving enamel integrity and improving its resistance [15]. The significant increase in phosphate and carbonate concentrations observed in the groups treated with MnO_BioS after the third bleaching session suggests that the remineralization process is occurring effectively, providing greater stability to the dental surface. This remineralization effect aligns with literature studies that demonstrate that the use of bioactive gels favors mineral deposition and improves the mechanical properties of enamel [19]. Moreover, the application of remineralizing agents not only prevents mineral loss during bleaching but also facilitates the recovery of surface hardness [33].

An in vitro study has shown that the pH increase of the MnO_BioS hydrogel to around 9.0 may explain the observed increase in %SHR in enamel during bleaching, as an acidic pH would promote demineralization [24]. Additionally, research on the synthesis of MnO_BioS indicates that, when in contact with simulated body fluid, the doped MnO promotes the formation of a silica‐rich layer and calcium phosphate precipitation, with a good capacity to form hydroxyapatite, even with moderate MnO doping [24]. These findings may explain enamel remineralization during bleaching and support the results observed in this study.

The results of this in situ study indicate that the addition of MnO_BioS to the bleaching gel with HP significantly enhances the effectiveness of dental bleaching. The use of intraoral palatal devices allowed the direct application of treatments in the oral environment, simulating real‐world conditions and highlighting the positive impact of the combination of MnO_BioS with HP on dental bleaching. However, it should be kept in mind that these findings are limited to the specific light protocol and bleaching regimen employed. Variations in the number of sessions, application time, or light parameters may alter the outcomes, necessitating caution when extrapolating these results.

Moreover, some limitations should be considered. First, future research is needed to evaluate the stability of remineralization effects over time to confirm the long‐lasting impact on enamel. Second, the long‐term safety of MnO_BioS use should be assessed to ensure there are no adverse effects on dental health or surrounding tissues. Additionally, studies comparing different concentrations of HP combined with bioactive agents could provide a broader understanding of the effectiveness of bleaching treatments. Finally, although bovine enamel exhibits slightly lower microhardness than human enamel, it has been extensively validated and widely employed in bleaching‐related research [34, 35, 36, 37, 38]. Recent evidence demonstrates comparable color change and HP penetration between bovine and human enamel, supporting its suitability for evaluating bleaching efficacy and associated physicochemical effects under controlled conditions [35, 36, 37, 39]. Nevertheless, inherent mechanical differences may influence the response of bovine enamel to chemical challenges, limiting direct clinical extrapolation. Therefore, although the present findings should be interpreted with appropriate caution, bovine enamel remains a reproducible and reliable substrate for comparative in situ investigations conducted under identical oral environments. Despite these limitations, the study reinforces the potential of adding MnO_BioS to the bleaching gel with 6%HP irradiated with violet LED light, surpassing the bleaching index when compared to 35%HP, while also protecting enamel integrity and calcium and phosphate concentrations on the dental surface.

## AUTHOR CONTRIBUTIONS

**Conceptualization**: Vanessa Cavalli, Rafael Dascanio, Marina Trevelin Souza. **Formal Analysis**: Rafael Dascanio, Marina Trevelin Souza, Camila Siqueira Silva Coelho, Matheus Kury, Edgar Dutra Zanotto. **Methodology**: Rafael Dascanio, Marina Trevelin Souza, Camila Siqueira Silva Coelho, Matheus Kury, Edgar Dutra Zanotto. **Original Draft Writing**: Rafael Dascanio, Vanessa Cavalli. **Writing‐Review & Editing**: Rafael Dascanio, Marina Trevelin Souza, Camila Siqueira Silva Coelho, Matheus Kury, Edgar Dutra Zanotto, Vanessa Cavalli.

## CONFLICT OF INTEREST STATEMENT

The authors declare no conflicts of interest.

## Data Availability

The data that support the findings of this study are available from the corresponding author upon reasonable request.
